# Supplementary figures and images for: Regulatory Role of Meox1 in Muscle Growth of Sebastes schlegelii
Source: Int J Mol Sci. 2024 Apr 29;25(9):4871. doi: 10.3390/ijms25094871 (PMC11084361; doi:10.3390/ijms25094871)

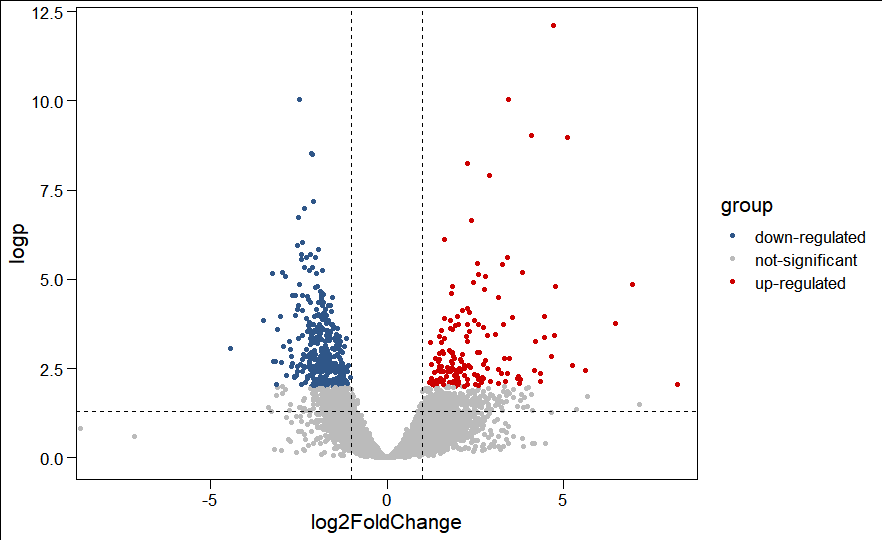

Supplement: Supplementary file 1 [file ijms-25-04871-s001.zip › Figure S1-Volcano plot of differentially expressed genes of G-40D samples .png]
